# Why Communities Should Be the Focus to Reduce Stigma Attached to COVID-19

**DOI:** 10.4269/ajtmh.20-1329

**Published:** 2020-11-30

**Authors:** Lydia Bologna, Katherine V. Stamidis, Sarah Paige, Roma Solomon, Filimona Bisrat, Anthony Kisanga, Samuel Usman, Ahmed Arale

**Affiliations:** 1 CORE Group Polio Project, Washington, District of Columbia;; 2 Global Health Security Agenda, CORE Group, Washington, District of Columbia;; 3 CORE Group Polio Project/India, Gurgaon, India;; 4 CORE Group Polio Project/Ethiopia, Addis Ababa, Ethiopia;; 5 CORE Group Polio Project/South Sudan, Juba, South Sudan;; 6 CORE Group Partners Project/Nigeria, Abuja, Nigeria;; 7 CORE Group Polio Project/Kenya and Somalia, Nairobi, Kenya

## Abstract

Since 1999, the CORE Group Polio Project (CGPP) has developed, refined, and deployed effective strategies to mobilize communities to improve vaccine uptake for polio (and other vaccine-preventable diseases such as measles) and conduct surveillance for infectious disease threats in high-risk, border, and hard-to-reach locations. CORE Group Polio Project teams have been called upon to address the COVID-19 pandemic, and, like with polio, the pandemic response is impacted by stigma in all areas of response, from health education, testing, contact tracing, and even treatment for infected individuals. The CGPP has reached back into its polio experience and is redeploying successful community engagement activities to address stigma as part of the COVID-19 response. Across country programs, community health volunteers communicate risk and behavior change at the household level by integrating health education and promotion activities with a focus on practical measures of COVID-19 prevention. Moreover, leveraging established and trusted partnerships with community networks and community leaders are providing lessons that can be adopted by the global community. The CGPP offers three overarching recommendations to curb stigma: 1) facilitating inclusive community engagement, 2) leveraging existing community networks and 3) cocreating with community leaders.

## INTRODUCTION

In a rare victory for global health this year, the WHO declared the absence of indigenous wild polio virus from its 47-nation African Region. Africa saw its last endemic case in 2016 in the state of Borno in northeastern Nigeria. Pakistan and Afghanistan are the last two endemic countries; both are experiencing spiking transmission. The outlook for polio eradication is complicated by explosive outbreaks of vaccine-derived poliovirus.^†^ These outbreaks have spilled into 22 countries and continue to climb, exacerbated by the WHO’s decision to suspend polio vaccination campaigns in 28 countries because of COVID-19.^1,2^

The CORE Group Polio Project (CGPP) operates with 11 international nongovernmental organizations (NGOs) and about 20 national and local NGOs in eight countries: India, Ethiopia, South Sudan, Nigeria, Kenya, Somalia, Uganda, and Afghanistan. The CGPP, funded by the United States Agency for International Development (USAID), is premised on the concept that disease outbreaks are best identified and interrupted at the community level.^3^ To that end, the CGPP has cultivated a strong volunteer corps to mobilize communities to serve as partners in advocating for the delivery of quality vaccination and health education, conducting community-based surveillance, promoting essential immunization, and tracing the vaccination status of key populations. As frontline workers, CGPP-trained volunteers are credible, highly valued, trusted, and well respected because they are from the target communities.^4^ When faced with reluctance associated with polio vaccination uptake or with stigma against vaccinators, community volunteers shift attitudes and behaviors through honest, informed, and compassionate interpersonal communication and two-way dialogue in local languages.^5^ Because of the reach of this massive volunteer corps, the CGPP was rapidly called upon to respond to the COVID-19 pandemic and the numerous forms of resistance undermining pandemic response, especially stigma.

## HEALTH IMPACTS OF STIGMA

Stigma is a Greek word that means to cut or burn the skin of criminals, slaves, or traitors to visibly mark them as blemished or morally polluted.^6^ Health-related stigma is not a new phenomenon. It has plagued those infected and affected by emerging and established infectious diseases for centuries. The mention of illnesses such as leprosy, the plague, tuberculosis, and more recently Ebola and HIV/AIDS conjures up notions of isolation, discrimination, fear, and aggression.^7^ Health-related stigma is defined as “the negative association between a person or group of people who share certain characteristics and aspecific disease.”^8^ Common drivers of stigma include fear of infection, misinformation, economic consequences of disease, lack of awareness, and socially constructed stereotypes.^8–12^ Experiences with stigma are typically defined at the individual level.^10,13^ However, the drivers and impacts of stigma reach beyond the individual. The Health Stigma and Discrimination Framework highlights the utility of an ecological approach by accounting for experiences and drivers at the individual, family, community, and policy levels, and incorporates important underlying factors including social norms and socioeconomic conditions.^12,14^ Whereas some drivers of stigma such as fear and misinformation appear to be universal, others are linked to country and community context such as social biases, anger, and distrust in the government. The effects of stigma and discrimination are far reaching and can affect mental and physical health, social cohesion, and economic systems, and worsen existing inequities in already marginalized communities.^15^ COVID-19 stigma impacts specific groups of people that vary by country, region, and community. These groups include healthcare workers, COVID-19 patients and people who have recovered, families of people infected with COVID-19, and, specific to the CGPP implementation sites, nomadic pastoralists, and other “outsiders.” The COVID-19 response from governments and NGOs has been dramatically impeded, further hindering efforts to halt the pandemic.

## COVID-19–RELATED STIGMA IN CGPP OPERATIONAL AREAS

**Figure 1.**
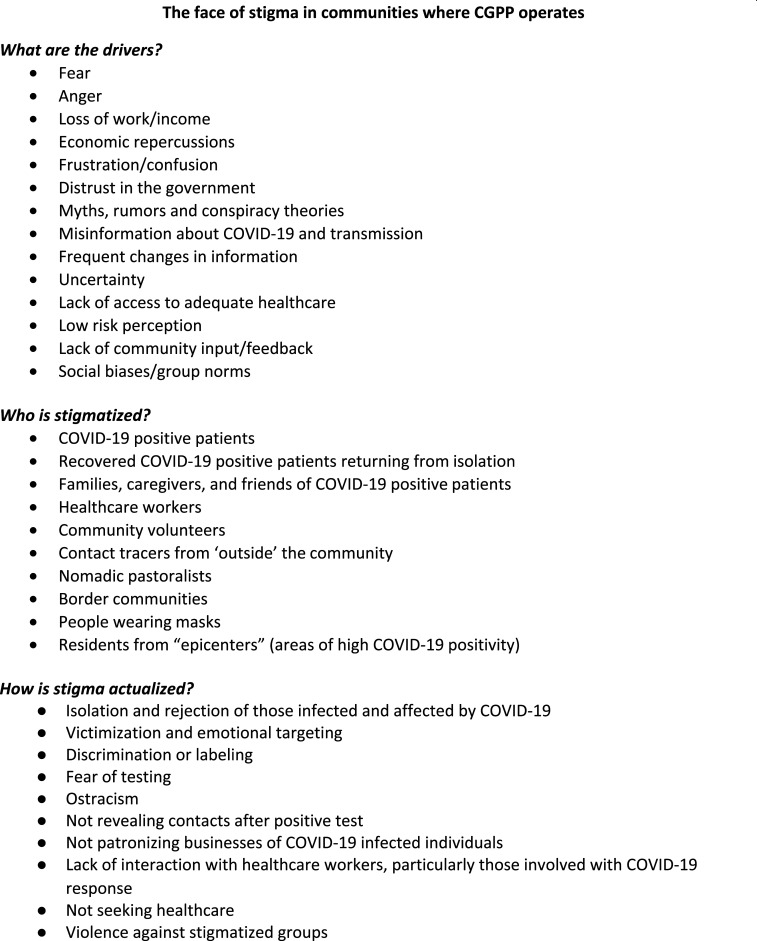
Stigma in CORE Group Polio Project (CGPP) communities at a glance.

The first cases of COVID-19 in CGPP implementation areas were reported between January 30 and April 5, 2020, and stigma quickly followed. Stigma impacts health education, testing, contact tracing, and even treatment for infected individuals. In India, a fruit vendor, his wife, and two adult children were the first with COVID-19 in their village. They fully recovered and returned home from an isolation center only to be boycotted by neighbors and relatives, leaving the family ostracized, isolated, and cutoff from their only source of income. In South Sudan, unfamiliar contact tracers who enter a village are faced with suspicion, beaten, or chased off. Figure 1 highlights the different manifestations of stigma, the various populations affected, and its consequences in CGPP areas.

## RECOMMENDATIONS TO ADDRESS STIGMA

The CGPP’s response to COVID-19 stigma is a natural outgrowth of community engagement strategies tested and strengthened over the past 20 years. Country teams have a depth of experience and capacity for transforming narratives of stigma to resilience.^16,17^ In CGPP implementation areas, the drivers of stigma have been fueled by the massive and complex body of information and misinformation, preexisting and renewed mistrust in, and anger toward the government/public sector, fear of the unknown, and implicit bias. These drivers are common across the globe, but what lies underneath these drivers is unique to each place, and therefore, addressing stigma requires a level of familiarity that is only accessible to individuals and groups who are part of the deeply woven fabric of each community.

**Figure 2.**
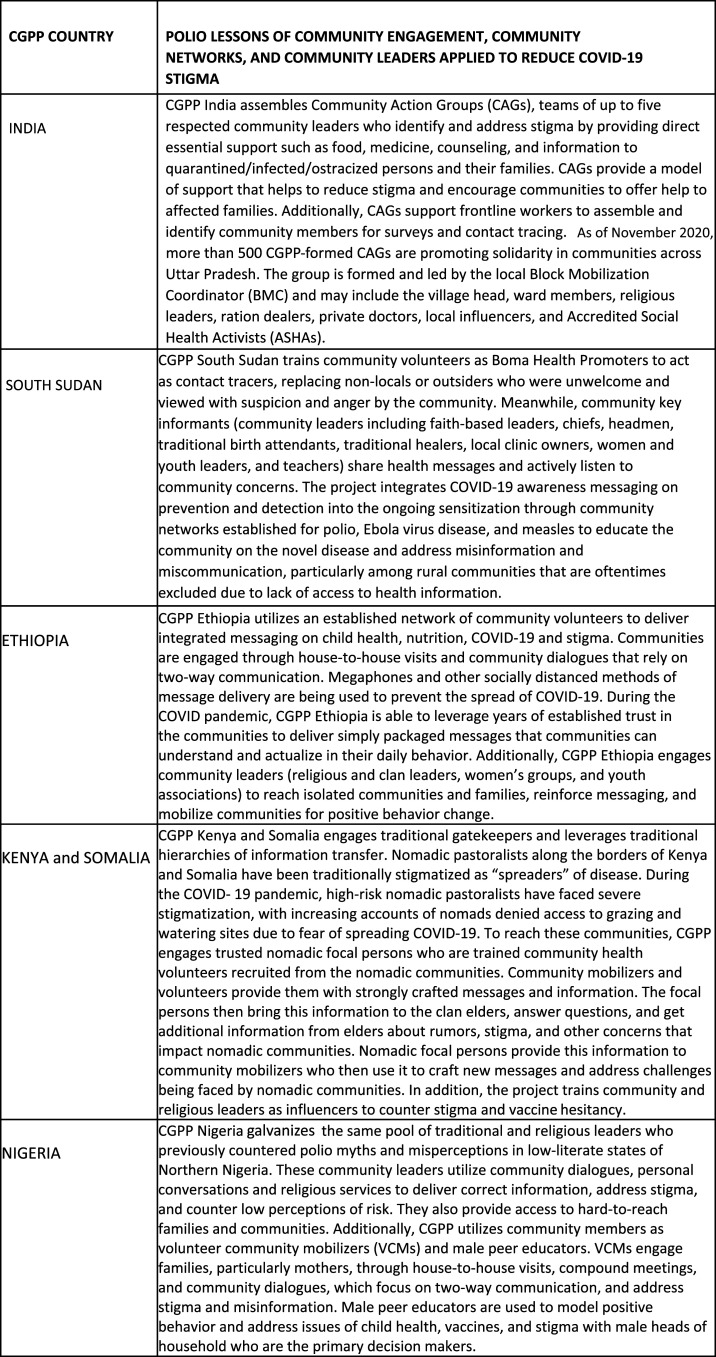
Spectrum of country-specific applications of polio lessons to COVID-19 stigma.

The CGPP has identified three overarching recommendations to address stigma. Although each recommendation must be contextualized to the country and community, all prioritize a genuine, bottom-up approach. Drawn from two decades of polio experience, these approaches are being used again to address and dismantle COVID-19 stigma. The three recommendations for action are as follows: 1) facilitating inclusive community engagement, 2) leveraging existing community networks, and 3) cocreating with community leaders. Figure 2 presents a spectrum of country-specific applications of polio lessons to curb COVID-19 stigma.

### Facilitating inclusive community engagement.

The CGPP is based on the premise of building community capacity through enabling participatory, inclusive community engagement by ensuring all voices are heard. In the context of the CGPP model of community engagement, communities that are most marginalized and vulnerable are also critical partners with trusted networks and influential leaders to understand, define, and achieve shared goals to empower families with control over their health and well-being.^18^ The project operates at the grassroots to mobilize high-risk populations to encourage community uptake of risk mitigation and behavior change recommendations. The CGPP has long recognized the importance of community-centric measures shaped by local and national NGOs that regularly partner with communities, enabling insights and perceptions, and countering negative associations often amplified by social norms while recognizing wider socioeconomic needs. This allows the project to swiftly detect issues related to stigma and to identify and implement appropriate, highly relevant response measures; ideally, this strategic approach aims to dismantle the narrative of “us versus them” in an attempt to coproduce a plan that is valued by all. Maximal input from at-risk communities should be a fundamental practice—the first step and not an afterthought; meaningful participation of communities, however, depends on collaboration with established, trusted partnerships.^5,19,20^

### Leveraging existing community networks.

A second recommendation gleaned from polio experience is to leverage existing community networks. Through 20 years of CGPP community engagement and partnership, the program has identified, trained, and supported a vast network of 21,067^2^ community volunteers, mobilizers, outreach workers, and informants comprising youth, women, pastoralists, and others, to support polio programming. Networks are long-term relationships among community volunteers, the public health sector, and the CGPP teams that partner to achieve health goals. These goals range from promotion of vaccination campaigns to mitigating misinformation and stigma.^21^ By working through that preexisting network, information is quickly shared, and feedback is quickly collected and used to adapt programming, implementation, and recommendations. The network is also a resource for sharing community priorities, challenges, and successful practices up to the global level to inform broader community engagement approaches and to elevate the voice of the community.^22,23^ The International Health Regulations’ monitoring tool (Joint External Evaluation 2.0) promotes the establishment and maintenance of community networks as part of a portfolio of activities required to fulfill capacities toward risk communication for outbreak response.^24^

### Cocreating with community leaders.

A third recommendation assembled from the polio experience is to use established relationships with respected community leaders to catalyze change. Through polio programming, the CGPP identified, trained, and engaged community gatekeepers and influencers to add momentum to social mobilization and community engagement, motivate positive behavior change, and create a pathway to community ownership. Gatekeepers have strong roots in communities and provide keen understanding of and connections to the most at risk. The CGPP engages religious leaders, village elders, nomadic pastoralist headmen, and traditional and clan leaders who work alongside community volunteers to provide access, resolve cases of vaccine hesitancy and noncompliance, and connect communities with services and information. The CGPP also leverages influencers who are trusted opinion leaders in their communities.^25^ They have the power to create and change community dialogues and drive conversations. When they speak, others listen. The CGPP engages religious, informal, and traditional leaders, traditional birth attendants, trade leaders, traditional healers, salon owners, and other influencers to work synergistically with networks of volunteers to reinforce and amplify messages, model positive behaviors, and address myths and misconceptions. Community leaders are uniquely positioned to identify, understand, and address the contextual drivers of stigma in their communities.

## CONCLUSION

The CGPP’s current response to COVID-19 stigma is heavily dependent on tapping highly collaborative strategies of community partnership. Grounded in trust and inclusion, the CGPP is now using these vital strategies for its pandemic response program and will deploy these again during the rollout of a COVID-19 vaccine. In anticipation of widespread vaccine hesitancy and resistance, the project’s response will be defined by experiences from polio work through maintaining and building on these enduring community-centric strategies. These approaches have found success by creating a climate of resilience in communities facing overlapping challenges.

## Footnotes

2 Based on Fiscal Year 2020 Quarter 3 program administrative data.

## References

[1] Independent Monitoring Board, 18th Report, 2020 Global Polio Eradication Initiative. Available at: http://polioeradication.org/wp-content/uploads/2020/08/20200816-IMB-18th-Report-FINAL.pdf. Accessed August 18, 2020.

[2] UNICEF and World Health Organization, 2020 Emergency Call to Action for Measles and Polio Outbreak Prevention and Response. Available at: https://polioeradication.org/news-post/unicef-and-who-call-for-emergency-action-to-avert-major-measles-and-polio-epidemics/. Accessed November 6, 2020.

[3] Global Preparedness Monitoring Board, 2019 From Words to Action: Towards a Community-Centered Approach to Preparedness and Response in Health Emergencies. Geneva, Switzerland: International Federation of Red Cross and Red Crescent Societies Available at: https://apps.who.int/gpmb/assets/thematic_papers/tr-5.pdf. Accessed September 1, 2020.

[4] Lewis J LeBan K Solomon R Bisrat F Usman S Arale A, 2020 The critical role and evaluation of community mobilizers in polio eradication in remote settings in Africa and Asia. Glob Health Sci Pract 8: 396–412. 33008854 10.9745/GHSP-D-20-00024 PMC7541117

[5] Solomon R, 2019 Involvement of civil society in India’s polio eradication program: lessons learned. Am J Trop Med Hyg 101 (Suppl 4): 15–20. 31760980 10.4269/ajtmh.18-0931 PMC6776100

[6] Stigma, Encyclopedia.com, 2020 The Oxford Pocket Dictionary of Current English. Available at: https://www.encyclopedia.com/humanities/dictionaries-thesauruses-pictures-and-press-releases/stigma. Accessed August 11, 2020.

[7] Hofstraat K van Brakel WH, 2016 Social stigma towards neglected tropical diseases: a systematic review. Int Health 8 (Suppl 1): 53–70. 26940310 10.1093/inthealth/ihv071

[8] Perry P Donini-Lenhoff F, 2010 Stigmatization complicates infectious disease management. AMA J Ethics 12: 225–230. 10.1001/virtualmentor.2010.12.3.mhst1-1003 23140873

[9] Fischer LS Mansergh G Lynch J Santibanez S, 2019 Addressing disease-related stigma during infectious disease outbreaks. Disaster Med Public Health Prep 13: 989–994. 31156079 10.1017/dmp.2018.157 PMC6889068

[10] Hatzenbuehler ML Phelan JC Link BG, 2013 Stigma as a fundamental cause of population health inequalities. Am J Pub Health 103: 813–821. 23488505 10.2105/AJPH.2012.301069 PMC3682466

[11] World Health Organization, 2020 Social Stigma Associated with COVID-19. Available at: https://www.who.int/docs/default-source/coronaviruse/covid19-stigma-guide.pdf. Accessed 11 August 2020.

[12] Stangl AL Earnshaw VA Logie CH Van Brakel W Simbayi L Barré I John D, 2019 The health stigma and discrimination framework: a global, crosscutting framework to inform research, intervention development, and policy on health-related stigmas. BMC Med 17: 31. 30764826 10.1186/s12916-019-1271-3 PMC6376797

[13] Goffman E, 1963 Stigma: Notes on the Management of Spoiled Identity. New York, NY: Simon & Schuster.

[14] Tam T, 2019 The Chief Public Health Officer's Report on the State of Public Health in Canada 2019: Addressing Stigma Towards a More Inclusive Health System. Available at: http://nccdh.ca/resources/entry/addressing-stigma-towards-a-more-inclusive-health-system. Accessed August 9, 2020.

[15] Weiss MG, 2008 Stigma and the social burden of neglected tropical diseases. PLoS Negl Trop Dis 2: e237. 18478049 10.1371/journal.pntd.0000237 PMC2359851

[16] Andrus J Perry H, 2019 Impact, innovation and inclusion of civil society organizations in polio eradication: the CORE Group Polio Project story. Am J Trop Med Hyg 101 (Suppl 4): 1–112. 10.4269/ajtmh.19-0529 PMC6776102 31760981

[17] Manoncourt E Nkowane B Aziz R, 2019 Comprehensive Assessment of CORE Group Polio Project: A Process Evaluation Perspective. Washington, DC: CORE Group Polio Project Available at: https://coregroup.org/wp-content/uploads/2020/03/CGPP-External-Eval_web.pdf. Accessed July 6, 2020.

[18] Perry H Solomon R Bisrat F Hilmi L Stamidis K Steinglass R Weiss W Losey L Ogden E, 2019 Lessons learned from the CORE Group Polio Project and their relevance for other global health priorities. Am J Trop Med Hyg 101 (Suppl 4): 107–112. 31760974 10.4269/ajtmh.19-0036 PMC6776095

[19] Losey L 2019 The CORE Group Polio Project: an overview of its history and its contributions to the global polio eradication initiative. Am J Trop Med Hyg 101 (Suppl 4): 4–14. 31760971 10.4269/ajtmh.18-0916 PMC6776098

[20] Unicef Communication for Development (C4D), 2020 Minimum Quality Standards and Indicators for Community Engagement. UNICEF: New York. Available at https://www.unicef.org/mena/reports/community-engagement-standards. Accessed May 7, 2020.

[21] Stamidis K Bologna L Bisrat F Tadesse T Tessema F Kang E, 2019 Trust, communication, and community networks: how the CORE Group Polio Project community volunteers led the fight against polio in Ethiopia’s most at-risk areas. Am J Trop Med Hyg 101 (Suppl 4): 59–67. 10.4269/ajtmh.19-0038 PMC6776093 31760978

[22] Tessema F Bisrat F Kidane L Assres M Tadesse T Asegedew B, 2019 Improvements in polio vaccination status and knowledge about polio vaccination in the CORE Group Polio Project implementation areas in pastoralist and semi-pastoralist regions in Ethiopia. Am J Trop Med Hyg 101 (Suppl 4): 52–58. 31760976 10.4269/ajtmh.19-0022 PMC6776097

[23] Arale A Lutukai M Mohamed S Bologna L Stamidis K, 2019 Preventing importation of poliovirus in the horn of Africa: the success of the cross-border health initiative in Kenya and Somalia. Am J Trop Med Hyg 101 (Suppl 4): 100–106. 31760979 10.4269/ajtmh.19-0040 PMC6776092

[24] World Health Organization, 2018 Joint External Evaluation Tool: International Health Regulations (2005), 2nd edition WHO: Geneva, Switzerland Available at: https://www.google.com/url?q=https://www.who.int/ihr/publications/WHO_HSE_GCR_2018_2/en/&sa=D&ust=1601389863716000&usg=AFQjCNEm_cIYrAV7TdIgYOdfD4arcNu9TA. Accessed September 15, 2020.

[25] Usman S Bologna L Stamidis K, 2019 The CORE Group Partners Project in North East Nigeria: community engagement strategies to combat skepticism and build trust for vaccine acceptance. Am J Trop Med Hyg 101 (Suppl 4): 68–73. 31760975 10.4269/ajtmh.19-0143 PMC6776099

